# ﻿*Hydrangeaxinfeniae* (Hydrangeaceae), a new species from Sichuan, China

**DOI:** 10.3897/phytokeys.238.114289

**Published:** 2024-02-02

**Authors:** Jian Ru, Wen-Bin Ju, Heng-Ning Deng, Bo Xu, Xiang Zhou, Chuan-Jiong Liu, Wei Huang

**Affiliations:** 1 CAS Key Laboratory of Mountain Ecological Restoration and Bioresource Utilization & Ecological Restoration and Biodiversity Conservation Key Laboratory of Sichuan Province, Chengdu Institute of Biology, Chinese Academy of Sciences, Chengdu 610041, Sichuan, China Chengdu Institute of Biology, Chinese Academy of Sciences Chengdu China; 2 Key Laboratory for Regional Plants Conservation and Ecological Restoration of Northeast Jiangxi, College of Life Science, Shangrao Normal University, Shangrao 334001, Jiangxi, China University of Chinese Academy of Sciences Beijing China; 3 University of Chinese Academy of Sciences, Beijing 100049, China Shangrao Normal University Shangrao China; 4 Management Bureau of Huagaoxi National Nature Reserve, Xuyong 646499, Sichuan, China Management Bureau of Huagaoxi National Nature Reserve Xuyong China

**Keywords:** *Hydrangea* sect. *Dichroa*, morphology, taxonomy

## Abstract

*Hydrangeaxinfeniae* W.B.Ju & J.Ru, a new species of Hydrangeaceae from Sichuan Province, China, is described and illustrated. The new species belongs to Hydrangeasect.Dichroa (Lour.) Y.De Smet & Samain, with its distinctive characteristic being the nearly superior ovary. It shares morphological similarities with *H.yaoshanensis* (Y.C.Wu) Y.De Smet & C.Granados, but can be distinguished by its hirsute trichomes densely covered on the branchlets, leaves, peduncles and pedicels, broadly elliptic to rectangular-elliptic leaf blade with nearly rounded base, coarse teeth leaf margins, 3–4 pairs of lateral veins, corymbose cyme with few and loose branches, lanceolate bract, the calyx tube and lobes margin with sparsely hirsute trichomes, adaxially glabrous and abaxially sparsely hirsute petal, outer whorl filaments are linear, inner ones are awl-shaped, glabrous styles, and the nearly superior ovary. *H.xinfeniae***sp. nov.** currently known from only three relatively small populations of the type locality, and its conservation status is assessed as Data Deficient (DD).

## ﻿Introduction

*Hydrangea* L. (Linnaeus 1753) had been treated to be a member of the tribe Hydrangeeae in Hydrangeaceae, comprising approximately 73 species distributed from Eastern to Southeastern Asia, as well as from Southeastern North America to Central America and Western South America (Huang et al. 1995; Wei and Bartholomew 2001). However, morphological and phylogenetic studies have indicated that *Hydrangea* is not monophyletic (Hufford 1995, 1997; Soltis et al. 1995; Ge 2003; Jacobs 2010; Samain et al. 2010; Zhang et al. 2021). Based on molecular phylogenetic analyses, De Smet et al. (2015) proposed the broad concept of *Hydrangea*, which encompasses *Hydrangeas. str.* and the remaining eight satellite genera within Hydrangeeae, including *Broussaisia* Gaudich., *Cardiandra* Siebold & Zucc., *Decumaria* L., *Deinanthe* Maxim., *Dichroa* Lour., *Pileostegia* Hook.f. & Thomson, *Platycrater* Siebold & Zucc., Schizophragma Siebold & Zucc., grouped as a section within Hydrangea*s. l.* Furthermore, *Platycrater* is merged into Hydrangeasect.Asperae (Rehder) Y.De Smet & Samain. The broad concept of *Hydrangea* has been supported by Yang (2022). In this study, we also adopt the broad concept of *Hydrangea*.

Hydrangeasect.Dichroa (De Smet et al. 2015) comprises 12 species, widely distributed in the tropical and subtropical regions of Southeast Asia, with only a few species extending to Pacific islands. In China, there are six species of this section, distributed from the southwestern to eastern regions (Huang 1987; Huang et al. 1995; Wei and Bartholomew 2001). In recent years, new species from this section have been discovered in China (Huang et al. 2018; Deshmukh and Shende 2021).

During field investigations in Huagaoxi National Nature Reserve of Shuwei Town, Xuyong County, Sichuan Province, an unknown population of *Hydrangea* was discovered. After conducting a comprehensive review of relevant taxonomic literature (Chun 1954; Huang 1987; Huang et al. 1995; Wei and Bartholomew 2001; Jacobs 2010; Huang et al. 2018) and meticulously examining voucher specimens from various herbaria (A, B, C, CAS, CDBI, E, IBK, IBSC, P, PE, K, KUN, L, NYBG, US), we have identified that it represents a new species within Hydrangeasect.Dichroa, exhibiting morphological similarity with *H.yaoshanensis* (De Smet et al. 2015). In this study, we provide a detailed morphological characterization of this species based on our field observations and a thorough examination of the holotype specimen, and describe it as a new species.

## ﻿Material and methods

The voucher specimens of the new species in this study were collected from the type locality, Huagaoxi National Nature Reserve, and are deposited in CDBI and KUN. The morphological description of characteristics of the new species was conducted through both living plants in the field and voucher specimens. The morphological measurements of the new species were based on living plants. We examined available online specimen images of Hydrangeasect.Dichroa species stored in A, B, C, CAS, CDBI, E, IBK, IBSC, P, PE, K, KUN, L, NYBG, and the US through the Chinese Virtual Herbarium (https://www.cvh.ac.cn/) and JSTOR Global Plants (https://plants.jstor.org/). Additionally, we compared the morphological characteristics of the new species with those of similar species, relying on online voucher specimen images and published literature (Huang 1987; Huang et al. 1995; Huang et al. 2018; Deshmukh and Shende 2021).

## ﻿Taxonomic treatment

### ﻿ Hydrangea xinfeniae

Taxon classificationPlantaeCornalesHydrangeaceae

W.B.Ju & J.Ru sp. nov.

2F35B923-3E53-5BA6-BF91-1CBA1DA040DF

urn:lsid:ipni.org:names:77335528-1

Figs 1 , 2 , 3

#### Diagnosis.

*Hydrangeaxinfeniae* can be distinguished from the morphologically similar species *H.yaoshanensis* by the presence of densely hirsute trichomes on branchlets, leaves, peduncles and pedicels; leaf blades that are broadly elliptic to rectangular-elliptic with a nearly rounded base and coarse teeth along the leaf margin, lateral veins 3–4 on each side of the midvein; a corymbose cyme with few, loosely arranged branches and lanceolate bract; calyx tube sparsely covered with hirsute trichomes, with only the edges of the lobes bearing such hairs; a glabrous adaxial surface of the petals, while the abaxial surface is adorned with scattered hirsute trichomes; outer whorl filaments are linear, inner ones are awl-shaped; styles are glabrous, and a nearly superior ovary.

#### Type.

China. Sichuan Province: Xuyong County, Shuiwei Town,Huagaoxi National Nature Reserve, growing on the moist soil under the broadleaved forest, 28°13′29.97″N, 105°36′40.39″E, alt. 1368 m, 22 Jul. 2023, *W.B.Ju & R.Jiang J–1290* (holotype: CDBI!; isotype: KUN!).

#### Description.

Shrub, 55–80 cm tall, slightly curved in the upper part, usually prostrate in the lower part. Branchlets densely covered with hirsute trichomes. Leaves papery, opposite; petioles 2–4.5 cm long, densely covered with hirsute trichomes; blades broadly elliptic to rectangular-elliptic, 6–8 cm long, 4–6 cm wide, covered with hirsute trichomes on both surfaces, denser on the abaxial surface, apex acute or shortly acuminate, base entire and nearly rounded, non-decurrent, margin with coarse teeth, midrib and lateral veins conspicuous, raised on the abaxial surface, 3–4 lateral veins on each side of the midvein, not reaching the leaf margin, extending obliquely. Inflorescence corymbose cymose, loosely few branches; bracts lanceolate, 3.5–4.5 cm long, 1.5–1.8 cm wide, covered with hirsute trichomes on both surfaces; peduncle 0.4–1.2 cm long, densely covered with hirsute trichomes. Flower buds ovate; pedicels ca. 2 cm long, covered with densely hirsute trichomes; calyx tube inverted conical, ca. 2 mm long, sparsely covered with hirsute trichomes, lobes 5–6, lanceolate, ca. 2 mm long, with sparse hirsute trichomes only on the margin; corolla blue, lobes 5–6, free, narrowly ovate-triangular, base flat, gradually narrowing towards the apex, ca. 6 mm long, ca. 2 mm wide, slightly inwardly curved at the apex to form a hook, glabrous adaxially, covered with scattered hirsute trichomes abaxially; stamens 10–12 in two whorls, the outer stamens alternate the petals, and the inner stamens opposite the petals; anthers ovoid, longitudinally split; outer whorl filaments are linear, the base sometimes slightly widens, 2.8–3 mm long, inner ones awl-shaped, slightly wider at the base, gradually narrowing upwards, 2.2–2.3 mm long; styles 3–6, ca. 3 mm long, glabrous, ovary nearly superior, with numerous ovules. Berry nearly spherical, 5.5–6 mm in diameter, sparsely covered with hirsute trichomes. Mature seeds not observed.

**Figure 1.**
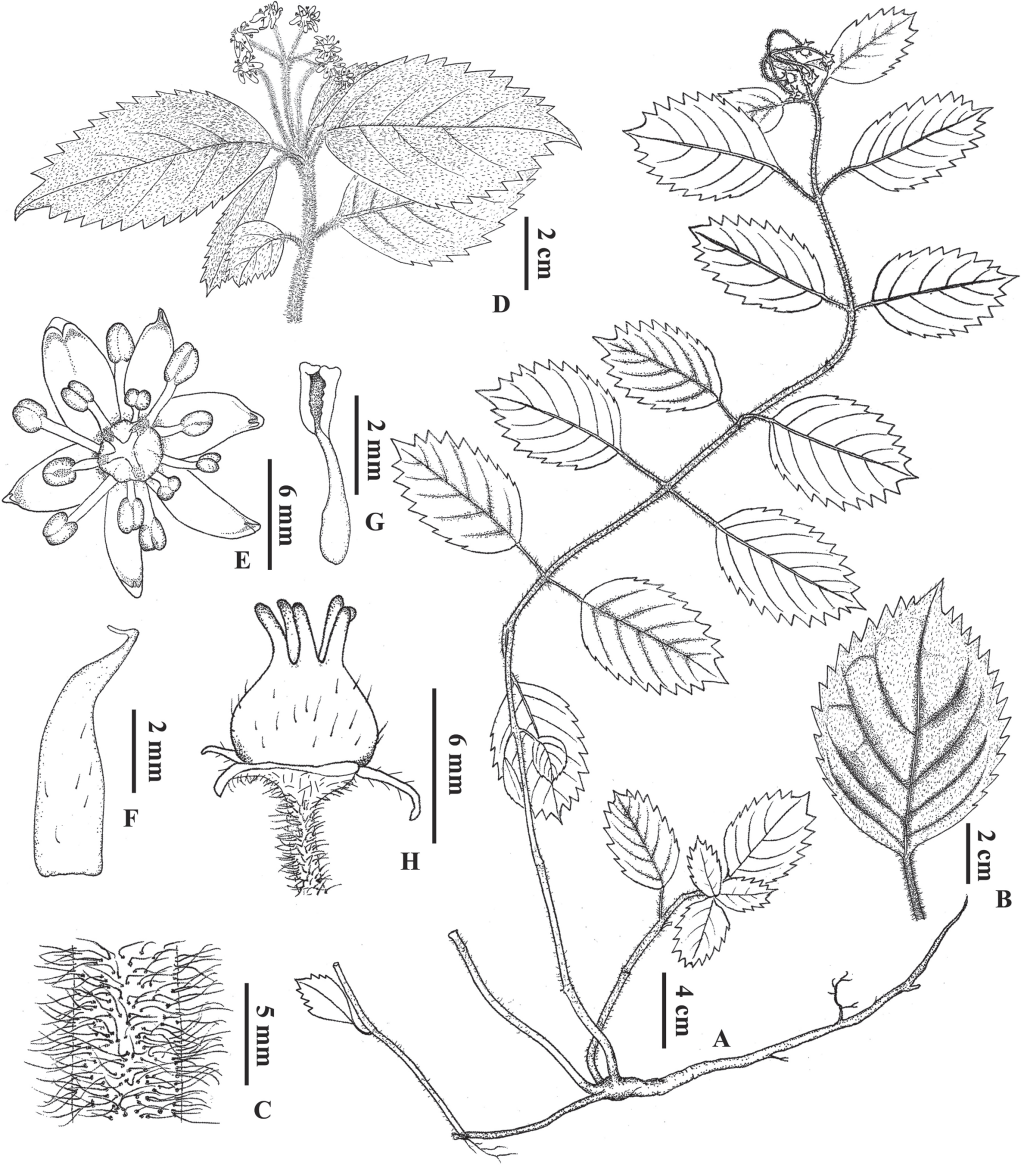
*Hydrangeaxinfeniae* W.B.Ju & J.Ru, sp. nov. **A** plant **B** leaf adaxial **C** twig **D** a branch with inflorescence **E** flower **F** petal **G** stamen **H** berry. Illustration drawn based on living plants (flower and berry) combined with specimens from the holotype by Zhenlong Liang.

#### Phenology.

Flowering from May to June; Fruiting from July to October.

#### Distribution and habitat.

*Hydrangeaxinfeniae* sp. nov. is found in its type locality, the Huagaoxi National Nature Reserve in Shuiwei Town, Xuyong County, Sichuan Province, China. It grows on moist soils under the broadleaved forest at an elevation of 1200–1300 meters.

**Figure 2.**
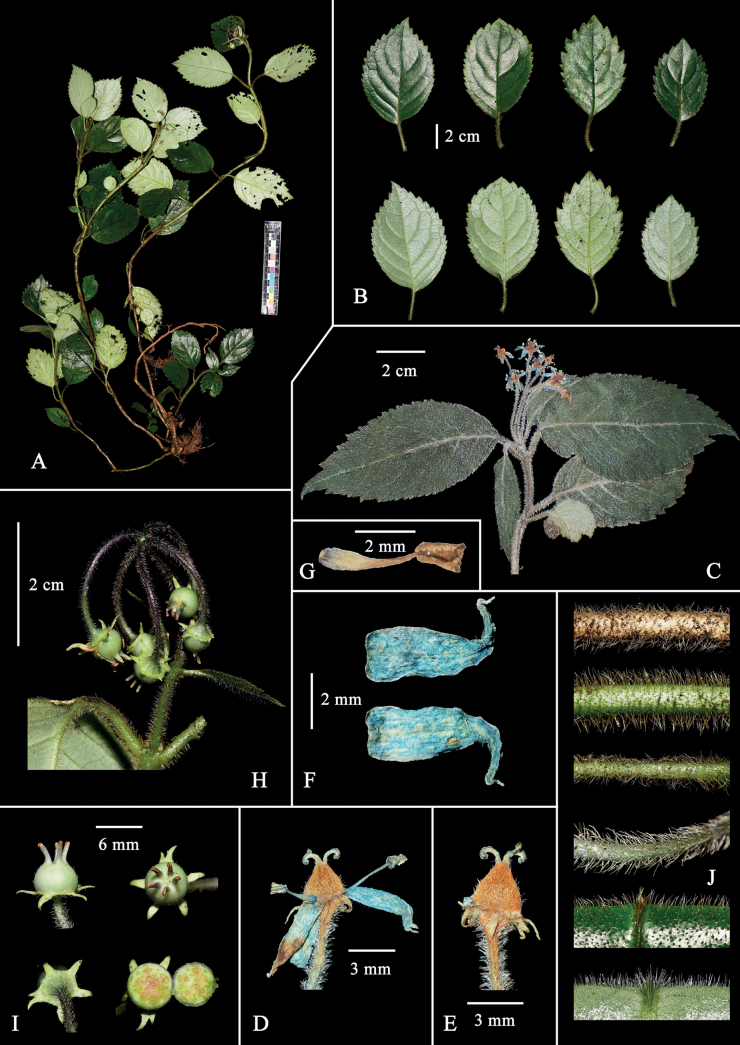
*Hydrangeaxinfeniae* W.B.Ju & J.Ru, sp. nov. **A** plant **B** leaves (upper: adaxial, lower: abaxial) **C** a branch with inflorescence **D, E** flower **F** petal (upper: adaxial, lower: abaxial) **G** stamen **H** infructescence **I** young berry **J** indumentum on different organs, from upper to lower, including old branchlet, young branchlet, petiole, carpopodium, leaf adaxial, and leaf abaxial.

#### Etymology.

The species epithet *xinfeniae* is in honor of Prof. Xinfen Gao, a distinguished female botanist who made significant contributions to the plant diversity survey of Huagaoxi National Nature Reserve.

#### Vernacular name.

Simplified Chinese: 信芬常山; Chinese pinyin: Xìnfēn cháng shān.

#### Additional specimens examined

**(paratypes).** China. Sichuan Province: Xuyong County, Shuiwei Town, Huagaoxi National Nature Reserve, 28°15′26.33″N, 105°28′58.89″E, alt. 1220 m, 5 Jun. 2015, *W.B.Ju J–441* (CDBI); ibid., 28°13′51.04″N, 105°37′3.80″E, alt. 1272 m, 10 Sep. 2023, *W.B.Ju & J.Ru J–1374* (CDBI).

#### Preliminary conservation assessment.

Based on the currently available survey data, only three relatively small populations have been discovered in Huagaoxi National Nature Reserve. Our knowledge regarding the status and distribution range of populations outside this area is limited. According to IUCN red list categories and criteria (IUCN 2022), the conservation status of the new species is temporarily assessed as Data Deficient (DD) due to insufficient available data. Further comprehensive surveys in similar environments and neighboring regions are necessary to provide a better assessment of the distribution and abundance of this species.

**Figure 3.**
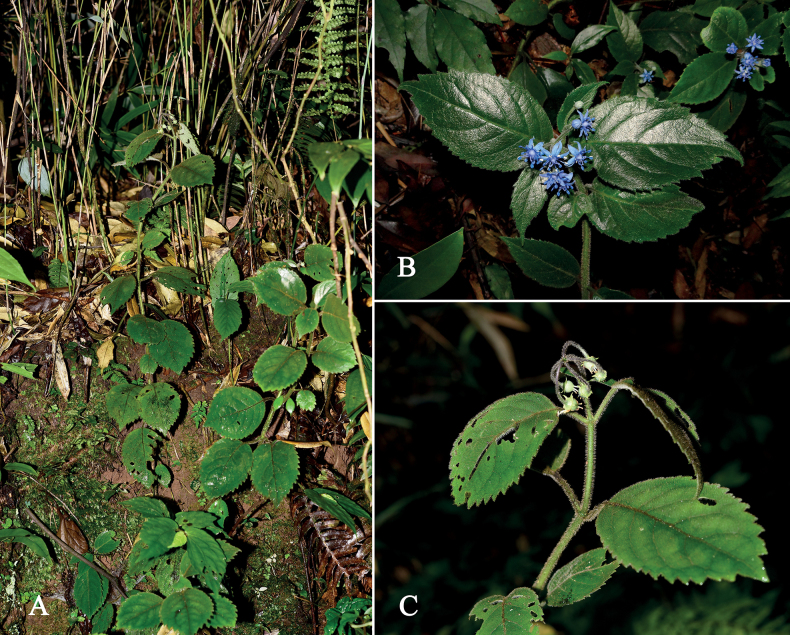
*Hydrangeaxinfeniae* W.B.Ju & J.Ru, sp. nov. in the wild **A** habit **B** a branch with inflorescence **C** fruiting branch.

## ﻿Discussion

Hydrangeasect.Dichroa is distinguished from related sections within *Hydrangea* by characteristics such as being an erect shrub, inflorescences with all fertile flowers, calyx lobes never petaloid, and fruit as berry. Based on a series of morphological characteristics, including shrub, opposite leaves, bisexual and isomorphic flowers, an inverted conical calyx tube attached to the ovary, petals with slightly inwardly curled tips forming hooks, outer whorl filaments are linear, inner whorl filaments awl-shaped, and separated styles, *Hydrangeaxinfeniae* sp. nov. is classified within the Hydrangeasect.Dichroa (Huang 1987; Huang et al. 1995; Wei and Bartholomew 2001; De Smet et al. 2015). Species within Hydrangeasect.Dichroa were previously treated as members of the genus *Dichroa* Lour. (Loureiro 1790) in Hydrangeeae. In China, Hydrangeasect.Dichroa comprises six known species, including *H.daimingshanensis* (Y.C.Wu) Y.De Smet & C.Granados, *H.febrifuga* (Lour.) Y.De Smet & C.Granados, *H.hirsuta* (Gagnep.) Y.De Smet & C.Granados, *H.mollissima* (Merr.) Y.De Smet & C.Granados, *H.yaoshanensis*, *H.yunnanensis* (S.M.Hwang) Y.De Smet & C.Granados, as well as one new species, *H.fistulosa* (G.H.Huang & G.Hao) U.B.Deshmukh & M.B.Shende (Huang et al. 1995; Huang et al. 2018; Deshmukh and Shende 2021). Among the aforementioned species distributed in China, *H.xinfeniae* sp. nov. exhibits morphological resemblances to *H.yaoshanensis* in having the shape of leaf blade, corymbose cymose, inverted conical calyx tube and spherical-shaped berry. However, *H.xinfeniae* sp. nov. can be distinguished by its dense hirsute trichomes on branchlets, leaves, peduncles, and pedicels, as well as its broadly elliptic to rectangular-elliptic leaf blades, nearly entire circular leaf basis, coarse teeth on the leaf margin, 3–4 lateral veins on each side, corymbose cyme with sparsely and loosely branched, calyx tube with sparse hirsute trichomes, lobes with sparse hirsute trichomes only on the margins, glabrous on the inner surface of the petals, scattered hirsute trichomes on the outer surface, outer whorl filaments are linear, inner ones are awl-shaped, styles are glabrous, and nearly superior ovary. Notably, the nearly superior ovary is a unique characteristic of *H.xinfeniae* sp. nov. compared to other species within Hydrangeasect.Dichroa. For a detailed comparison of features, please refer to Table 1.

**Table 1.** The comparison of morphological characters of *Hydrangeaxinfeniae* sp. nov. and *H.yaoshanensis*.

Characters	*H.xinfeniae* sp. nov.	*H.yaoshanensis* (Huang et al. 1995; Wei and Bartholomew 2001)
Habit	shrub, 55–80 cm tall	subshrub, up to 30 cm tall
Indumentum	branchlets, leaves, peduncles and pedicels densely covered with hirsute trichomes	branchlets, petioles, veins, and inflorescences covered with crisped pubescence and slightly pellucid hirsute trichomes
Leaf blade	broadly elliptic or rectangular-elliptic	elliptic or ovate-elliptic
	leaf base with nearly rounded, entire margins	leaf base cuneate or gradually narrowing, entire margins
	leaf margin with sparse coarse teeth	leaf margin serrate
	3–4 lateral veins on each side of midvein	5–11 lateral veins on each side of midvein
Inflorescence	corymbose cyme, loose, bract lanceolate	corymbose cyme, aggregate
	peduncle 0.4–1.2 cm	peduncle 0.5–1 cm
Pedicel	ca. 2 cm	ca. 5 mm
Calyx	calyx tube sparsely covered with hirsute trichomes	calyx tube densely covered with crisped pubescence and hirsute trichomes
	lobes ca. 2 mm long, with sparse hirsute trichomes only on the margins	lobes ca. 2.5–4 mm long, densely covered with hirsute trichomes on the outer and upper inner surfaces
Petal	glabrous on the inner surface, scattered hirsute trichomes on the outer surface	both surfaces densely covered with hirsute trichomes or without hairs on the inner surface
Stamen	outer whorl filaments are linear, inner ones are awl-shaped	filaments filiform
Pistil	styles glabrous	styles sparsely covered with hirsute trichomes at the lower part
	ovary nearly superior	ovary subinferior

## Supplementary Material

XML Treatment for Hydrangea xinfeniae

## ﻿Additional information

### Author ORCIDs

Jian Ru https://orcid.org/0000-0003-1268-3243

Wen-Bin Ju https://orcid.org/0000-0002-5665-6290

Heng-Ning Deng https://orcid.org/0000-0002-0581-0649

Bo Xu https://orcid.org/0000-0003-3507-9321

### Data availability

All of the data that support the findings of this study are available in the main text.
